# Outcome of patients treated with molecular adsorbent recirculating system albumin dialysis: A national multicenter study

**DOI:** 10.1002/jgh3.12359

**Published:** 2020-05-17

**Authors:** Christophe Camus, Clara Locher, Faouzi Saliba, Bernard Goubaux, Agnès Bonadona, Laurence Lavayssiere, Catherine Paugam, Alice Quinart, Olivier Barbot, Sébastien Dharancy, Bertrand Delafosse, Nicolas Pichon, Hélène Barraud, Arnaud Galbois, Benoit Veber, Sophie Cayot, Bruno Souche

**Affiliations:** ^1^ Service de Reanimation medicale, Hôpital Pontchaillou Rennes France; ^2^ Laboratoire de Pharmacologie, Centre d'Investigation Clinique INSERM 1414 Rennes France; ^3^ AP‐HP Hôpital Paul Brousse Centre Hépato‐biliaire Villejuif France; ^4^ Service d'Anesthesie Reanimation, Hôpital de l'Archet II Nice France; ^5^ Service de Reanimation medicale, Hôpital La Tronche Grenoble France; ^6^ Service de Nephrologie, Hôpital Rangueil—Larrey Toulouse France; ^7^ Service d'Anesthesie Reanimation chirurgicale, Hôpital Beaujon Clichy France; ^8^ Service d'Anesthesie Reanimation, Hôpital Pellegrin Bordeaux France; ^9^ Service de Reanimation medicale, Hôpital Jean Minjoz Besançon France; ^10^ Service d'Hepatologie, Hôpital Claude Huriez Lille France; ^11^ Service d' Anesthesie Reanimation chirurgicale, Hôpital Edouard Herriot Lyon France; ^12^ Service de Reanimation medicale, Hôpital Dupuytren Limoges France; ^13^ Service d'Hepato‐gastroenterologie, Hôpital de Brabois Vandoeuvre‐les‐Nancy France; ^14^ Service de Reanimation medicale, Hôpital Saint‐Antoine Paris France; ^15^ Service de Reanimation chirurgicale, Hôpital Charles Nicolle Rouen France; ^16^ Service de Reanimation, CHU Estaing Clermont‐Ferrand France; ^17^ Service d'Anesthesie reanimation, Hôpital Saint‐Eloi Montpellier France

**Keywords:** liver transplantation, MARS albumin dialysis, outcome, retrospective study

## Abstract

**Background and Aim:**

The molecular adsorbent recirculating system (MARS) is the most widely used device to treat liver failure. Nevertheless, data from widespread real‐life use are lacking.

**Methods:**

This was a retrospective multicenter study conducted in all French adult care centers that used MARS between 2004 and 2009. The primary objective was to evaluate patient survival according to the liver disease and listing status. Factors associated with mortality were the secondary objectives.

**Results:**

A total of 383 patients underwent 393 MARS treatments. The main indications were acute liver failure (ALF, 32.6%), and severe cholestasis (total bilirubin >340 μmol/L) (37.2%), hepatic encephalopathy (23.7%), and/or acute kidney injury–hepatorenal syndrome (22.9%) most often among patients with chronic liver disease. At the time of treatment, 34.4% of the patients were listed. Overall, the hospital survival rate was 49% (95% CI: 44–54%) and ranged from 25% to 81% depending on the diagnosis of the liver disease. In listed patients *versus* those not listed, the 1‐year survival rate was markedly better in the setting of nonbiliary cirrhosis (59% *vs* 15%), early graft nonfunction (80% *vs* 0%), and late graft dysfunction (72% *vs* 0%) (all *P* < 0.001). Among nonbiliary cirrhotic patients, hospital mortality was associated with the severity of liver disease (HE and severe cholestasis) and not being listed for transplant. In ALF, paracetamol etiology and ≥3 MARS sessions were associated with better transplant‐free survival.

**Conclusion:**

Our study suggests that MARS should be mainly used as a bridge to liver transplantation. Survival was correlated with being listed for most etiologies and with the intensity of treatment in ALF.

## Graphical abstract

Because the long‐term outcome of patients treated with the molecular adsorbent recirculating system (MARS) in the real life has not been well assessed, we performed a retrospective multicenter study in all French adult care centers that used MARS between 2004 and 2009. The hospital survival rate was 49% (95% CI: 44–54%) and varied from 25% to 81% depending on the diagnosis of the liver disease and the 1‐year survival rate was markedly better in listed *versus* not listed patients in the setting of non‐biliary cirrhosis (59% *vs* 15%), early graft non‐function (80% *vs* 0%) and late graft dysfunction (72% *vs* 0%) (all *P* < 0.001). In acute liver failure, paracetamol etiology and ≥3 MARS sessions were associated with higher transplant‐free survival.

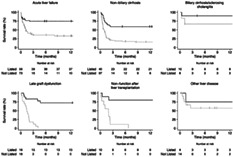

## Introduction

The majority of patients with advanced chronic liver disease does not receive liver transplantation (LTx). In Europe, the prevalence of cirrhosis has been estimated to be 26–133 per 100 000 inhabitants depending on the country, with around 170 000 deaths per year.^1^ In contrast, only 5000–8000 liver transplants per year were performed from 2000 to 2012.^2^ Due to this disparity, extracorporeal devices have been developed to support the failing liver in patients with chronic or acute liver diseases. Albumin dialysis using the molecular adsorbent recirculating system (MARS), which combines liver detoxification and renal replacement therapy, is the most frequently used extracorporeal liver support technique. MARS was developed from 1993 in order to remove albumin‐bound toxins^3^ or drugs with high affinity to albumin and began to be applied in the clinical setting in the late 1990s. Initial randomized controlled trials showed improved survival in patients with decompensated cirrhosis and type 1 hepatorenal syndrome,^4^ severe cholestasis (total bilirubin >20 mg/dL [>340 μmol/L]),^5^ or advanced hepatic encephalopathy.^6^

Besides chronic decompensated end‐stage liver disease, severe cholestasis, or intractable pruritus, MARS has also been used in other situations of liver failure, such as acute liver failure (ALF), liver failure after major hepatectomy, or after LTx.^7^, ^8^ MARS has been used mainly in the intensive care unit (ICU) for patients with organ dysfunctions associated with liver failure. Extensive data on the use of MARS therapy in real life and on long‐term outcomes are lacking. We therefore conducted a multicenter retrospective study to determine the indications for MARS treatments in the ICU and the outcome of patients according to liver disease diagnosis and to find whether or not they were listed for transplant (the RETROMARS study). This study reflects the real‐life use of MARS technique as a rescue therapy in a transitional period, before (2004‐March 2007) and after (March 2007–2009) the implementation of the Model of End‐stage Liver Disease (MELD) score in the French system for liver graft allocation.

## Patients and Methods

### *Objectives of the study*

The aims of the study were first to review the indications for MARS therapy reported by the physicians in charge of the patients at the time of treatments and, second, to assess hospital, 1‐year, and long‐term survival according to the underlying diagnosis.

### *Recruitment of participating centers*

This was a retrospective multicenter study of all patients treated with MARS over the period 2004–2009. All French hospitals where the MARS technique was regularly performed were asked to participate in the study. Patients who had undergone MARS therapy in an adult care setting in the course of the 5‐year study period were eligible. Among the physicians in charge of the patients, one main investigator was designated in each center. A clinical research file was sent to investigators and was to be completed for each patient treated. The study was approved by the National Research Consultative Committee and the Institutional Ethics Committee.

### *Variables recorded in the clinical research file*

Characteristics of hospital stay, indications for MARS therapy, main diagnosis of the underlying disease, type of dialysis device, characteristics and number of sessions performed per treatment, registration on the national transplant waiting list, LTx, and outcome were assessed. In addition, reasons for not being listed for LTx, time and cause of death, and last follow‐up were collected in order to calculate the longest possible survival.

### *End‐points*

Indications for MARS therapy confirmed by investigators were classified. For each diagnosis category, indications were compared according to the waiting list status (listed, not listed). Hospital survival was assessed for each diagnostic category. Follow‐up after hospital discharge was reported at the longest available follow‐up. One‐year and long‐term survival were calculated separately for each diagnosis.

### *Statistical analysis*

The hospital survival rate was calculated with the 95% confidence interval (95% CI) using the Clopper‐Pearson method, and percentages were rounded to the nearest percent unit. For the two main diagnostic categories, variables associated with the hospital outcome were identified by using multivariate analysis and logistic regression. Total follow‐up was calculated from the date of admission to the hospital to latest follow‐up after hospital discharge. Patients surviving at hospital discharge who were lost to follow‐up were censored at the time of hospital discharge. One‐year and long‐term survival were calculated using the Kaplan–Meier estimator in each diagnostic category and were compared between the listed patients and those not listed. All tests were two‐sided, and a *P*‐value ≤0.05 was considered statistically significant.

## Results

### *Participating centers and treatments*

Of the 25 centers using MARS albumin dialysis among adults, 16 participated in the study. All centers were University‐affiliated tertiary care hospitals with a Hepatology unit,14 were also liver transplant centers, and the 2 centers that were not were connected to a liver transplant center. A total of 383 patients were enrolled in the study and underwent 393 MARS treatments in the course of 389 hospital stays. Only 14 treatments (3.6%) were performed in the two centers where LTx was not performed. Patient inclusion in a clinical trial was reported for 34 of the treatments (8.7%).

MARS treatment consisted of one or, most of the time, two or more albumin dialysis sessions performed every day or every other day and lasting 8 h (minimum duration 5 h). The 393 treatments totaled 1091 MARS sessions. The median number of sessions per treatment was 3 (IQR 2–3; range 1–11). At our request, the manufacturer (Gambro Hospal Society, Meyzieu, France) provided data on the use of MARS kits by each French center over the study period. Consequently, we were able to estimate that the number of MARS sessions performed in the 16 participating centers represented 78.5% of all MARS sessions performed in the 25 French adult care centers during the study period. Of the 393 treatments, 344 (87.5%) were performed in the ICU. The MARS monitor was combined either with a continuous renal replacement therapy device for 873 sessions (80.0%: PRISMA® [*n* = 796], PRISMAFLEX® [*n* = 14] [Gambro]; AQUARIUS® [*n* = 63], [Fresenius Medical Care, Fresnes, France]) or with a hemodialysis generator for 218 sessions (20.0%, mostly AK100/AK200, Gambro [*n* = 170]).

### *Patients*

The median age was 50 years (interquartile range [IQR] 41–58 years; range 13–82 years). The main diagnoses for the underlying disease are shown in Table 1. The most frequent diagnostic categories were nonbiliary cirrhosis (35.1%), ALF (32.8%) and posttransplant complications (15.4%: nonfunction after LTx [5.1%], late graft dysfunction [10.4%]). Conditions other than liver disease (encephalopathy, hyperammonemia, drug intoxication) were rare (2.3%). At the time of MARS treatment, 34.4% of all cases were listed for transplant. The proportions on the waiting list varied according to the diagnosis: 26.8% (nonbiliary cirrhosis), 50% (primary biliary cholangitis/primary sclerosing cholangitis), 23.8% (other chronic liver diseases), 55% (nonfunction after LTx), 41.5% (late graft dysfunction), 43.4% (ALF), 0% (liver failure after hepatectomy for cancer), and 6.3% (other diagnosis) (*P* < 0.001). Reasons for not being listed were generally major comorbidities, advanced age, or the perception that the patient was “too ill.” In alcohol‐related cirrhosis, the main reason for not listing was the failure of alcohol withdrawal. Consequently, only 21.8% of these patients were on the waiting list during their hospital stay. Among the listed patients, 86 (65.6%) were transplanted during their hospital stay (Table S1).

**Table 1:** Main diagnosis in 393 MARS treatments

	All	Listed/not listed
No. (percentage)		
Nonbiliary cirrhosis	138 (35.1)	37/101
Alcohol	101	22/79
HCV	22	10/12
Other virus	7	2/3
Autoimmune	5	1/4
Other	3	2/1
Biliary cirrhosis/sclerosing cholangitis	16 (4.1)	8/8
Primary biliary cirrhosis	5	3/2
Secondary biliary cirrhosis	5	3/2
Primary sclerosing cholangitis	5	2/2
Other biliary	1	0/1
Other chronic liver disease	21 (5.3)	5/16
Cholestasis due to various causes	9	0/9
Nodular regenerative hyperplasia	3	3/0
Hepatitis	2	0/2
Liver metastases	3	0/3
Other†	4	2/2
Nonfunction after liver transplantation	20 (5.1)	11/9
Late graft dysfunction	41 (10.4)	17/24
Liver failure after nontransplant liver surgery‡	12 (3.1)	0/12
Acute liver failure	129 (32.8)	56/73
Other diagnosis	16 (4.1)	1/15
Jaundice/cholestasis, unknown	4	1/3
Liver failure after nonhepatic surgery	2	0/2
Pruritus, unknown	1	0/1
Encephalopathy	1	0/1
Hyperammonemia	2	0/2
Drug intoxication**§**	6	0/6

† Nephronophthisis (1), portal vein thrombosis (1), Wilson's disease (1), not specified (1).

‡ Liver failure after hepatectomy (malignant liver tumor).

§ Calcium blockers (3), valproate (2), chloroquine (1).

### *Recorded indications for the 393 MARS treatments*

**Figure 1:**
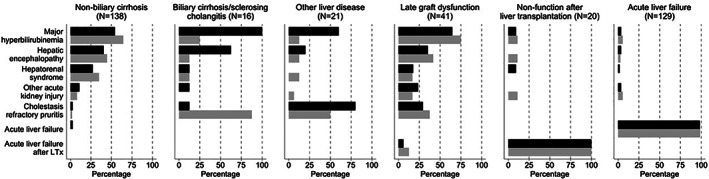
Indications for MARS therapy among cases listed for transplantation and cases not listed at the time of treatment according to the diagnosis. The indications for MARS are on the Y axis on the left part of the figure. Percentages of cases for listed and not listed cases are shown by black horizontal bars and gray horizontal bars. A single case can have more than one indication. In primary biliary cirrhosis/cholangitis, hyperbilirubinemia was more frequent among listed cases (*P* = 0.007), and refractory pruritus was more frequent among those not listed (*P* = 0.01). Details for numbers, percentages, and 95% confidence intervals are provided in the supporting information Table 2. (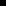) Listed; (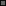) Not listed.

Irrespective of the diagnosis, the reasons for MARS therapy were classified into nine main indications (Table S2). The most frequent indications were major hyperbilirubinemia (37.2%, total bilirubin ≥340 μmol/L), ALF (32.6%), hepatic encephalopathy (HE, 23.7%), and acute kidney injury (AKI, 22.9%: hepatorenal syndrome [HRS, 15.3%]; non‐HRS AKI [7.6%]). MARS was more rarely performed for intractable pruritus (9.9%) and as an adjunctive treatment for hepatic failure after LTx (6.1%) or major hepatectomy (3.8%), and it was infrequently used for drug intoxication (1.5%). Although alcoholic hepatitis was reported to be an indication for MARS in 12.5% of treatments, it was not classified as a separate indication because all these patients also had associated major hyperbilirubinemia, HE, and/or AKI‐HRS syndrome. There was more than one indication for 37.2% of the treatments. ALF was reported as a full‐fledged indication for 92.2% of treatments of the patients finally diagnosed with ALF. Except for primary biliary cirrhosis/cholangitis, there were no major differences in the indications for MARS therapy between the patients listed for transplant and those not listed (Fig. 1).

### *Hospital, 1‐year, and long‐term survival according to the main diagnosis*

**Figure 2:**
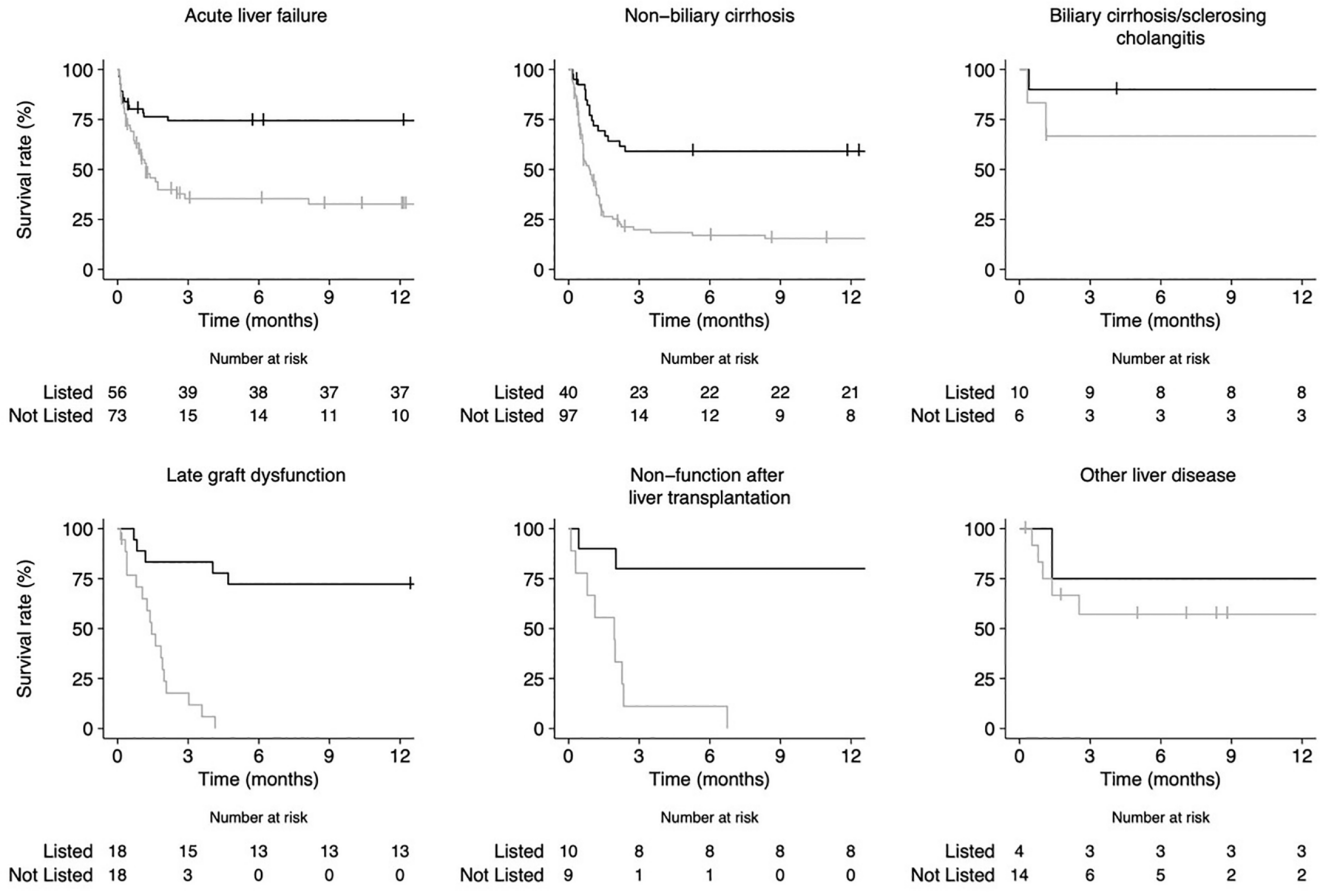
One‐year survival for listed patients compared to those who were not listed according to the diagnosis. Survival rates are expressed as percentages (95% confidence interval), listed *versus* not listed: acute liver failure: 75% (64–87%) *versus* 33% (22–48%), *P* < 0.0001; nonbiliary cirrhosis: 59% (45–77%) *versus* 15% (9–26%), *P* < 0.0001; biliary cirrhosis/sclerosing cholangitis: 90% (73–100%) *versus* 67% (38–100%), *P* = 0.26; late graft dysfunction: 72% (54–96%) *versus* 0, *P* < 0.0001; nonfunction after liver transplantation: 80% (59–100%) *versus* 0, *P* = 0.00045; other liver disease: 75% (43–100%) *versus* 57% (35–94%), *P* = 0.51. (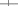) Listed; (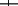) Not listed

Overall, the hospital survival rate was 49% (95% CI: 44–54%) and varied from 25% to 81% according to the diagnosis (Table 2). Among the 185 patients who were alive at hospital discharge, 32 patients were lost to follow‐up. Most of them were not eligible for LTx or had recovered from ALF during the hospital stay (Table S3). Ten patients with pre‐existing chronic liver disease were listed for transplant after hospital discharge. One‐year survival curves among patients listed and not listed are shown in Figure 2. Overall, to be listed was associated with higher survival rates in all diagnostic categories.

**Table 2:** Hospital survival in 389 stays

	No. who survived/total (% [95% CI])					
Main diagnosis	All patients		Not listed		Listed	
Chronic liver disease						
Nonbiliary cirrhosis	52/137	(38 [30–47])	32/101	(32 [23–42])	20/36	(56 [38–72])*
Biliary cirrhosis/sclerosing cirrhosis	13/16	(81 [54–96])	6/8	(75 [35–97])	7/8	(88 [47–100])
Other liver disease	15/21	(71 [48–89])	11/16	(69 [41–89])	4/5	(80 [28–99])
Liver failure after transplantation						
Nonfunction after liver transplantation	8/19	(42 [20–67])	0/9	(0 [0–34])	8/10	(80 [44–97])**
Late graft dysfunction	17/39	(44 [28–60])	7/24	(29 [13–51])	10/15	(67 [38–88])*
Liver failure after nontransplant liver surgery	3/12	(25 [5–57])	3/12	(25 [5–57])		
Acute liver failure	75/129	(58 [49–67])	33/73	(45 [34–57])	42/56	(75 [62–86])**
Other	8/16	(50 [25–75])	7/15	(47 [21–73])	1/1	(100 [25–100)
Total	191/389	(49 [44–54])	99/258	(38 [32–45])	92/131	(70 [62–78])**

*P < 0.05; **P < 0.001.

#### *Nonbiliary cirrhosis*

Among patients with nonbiliary cirrhosis, the overall hospital survival rate was 38% (30–47%) and was significantly higher among patients who were on the transplant list (56% [38–72%] than among those not listed (32% [23–42%], *P* = 0.01). In a multivariate logistic regression, independent risk factors for hospital death were not being on the transplant list (OR = 3.04 [95% CI 1.24–7.46], *P* = 0.02), MARS indication for HE (OR 2.94 [1.22–7.09], *P* = 002), and MARS indication for major hyperbilirubinemia (OR 2.44 [1.09–5.46], *P* = 0.03) (Table 3).

**Table 3:** Risk factors for hospital death in nonbiliary cirrhosis

	Univariate			Multivariate		
Variable	Odds ratio	95% CI	*P*‐value	Odds ratio	95% CI	*P*‐value
MARS indication for hepatic encephalopathy	4.33	1.99–9.35	<0.001	2.94	1.22–7.09	0.02
MARS indication for hyperbilirubinemia	3.68	1.77–7.63	<0.001	2.44	1.09–5.46	0.03
Nonlisted	2.70	1.24–5.88	0.01	3.04	1.24–7.46	0.02
Acute alcoholic hepatitis	0.42	0.21–0.86	0.02			
Hepatorenal syndrome	2.49	1.12–5.49	0.03			
Age (year)	1.03	0.995–1.07	0.09			

Among listed patients, 1‐year and 5‐year survival rates were 59% (45–77%) and 49% (35–69%), respectively. Among those not listed, the 1‐year survival rate was 15% (9–26%; *vs* listed: *P* < 0.0001), and 5‐year survival was not assessable (4‐year survival rate was 8% [3–33%]).

#### *Acute liver failure*

For ALF, the median number of sessions performed was 2 (IQR 1–3, range 1–8). Thirty‐eight patients (29%) were transplanted during their ICU stay. Fewer sessions were performed among the patients who underwent LTx (1 [1, 2]) compared to those not transplanted (2 [1–3], *P* < 0.001). Overall, hospital survival was 58% (50–66%) and was higher among listed patients (75% [62–86%]) than among those not listed (45% [34–57%], *P* < 0.001). Among the 56 patients who were listed, 18 were not transplanted: 9 improved and survived (50% [26–74%]), and 9 died while being on the waiting list. The difference in age between the patients who survived and those who died was not statistically significant (years of age, 44 [31–56] *vs* 49 [36–57], *P* = 0.13). To be listed with the “High‐emergency” priority was the only variable associated with hospital survival (OR 3.46 [1.61–7.45], *P* = 0.002). After adjustment for age, ALF etiology, number of sessions, and study year, the result was similar (OR 3.74 [1.67–8.40], *P* = 001). Transplant‐free survival was 33% (25–41%). After adjustment for age in multivariate analysis, ≥3 MARS sessions performed, and paracetamol etiology was associated with spontaneous survival (Table 4). The results did not change when patients who had a length of stay in ICU ≤72 h and were unlikely to receive three MARS sessions were excluded (Table S4).

**Table 4:** Variables associated with hospital spontaneous survival in acute liver failure

	Univariate			Multivariate		
Variable	Odds ratio	95% CI	*P*‐value	Odds ratio	95% CI	*P*‐value
Listed with the “High emergency” priority	0.23	0.10–0.54	<0.001	0.25	0.10–0.63	0.003
≥3 MARS sessions	2.69	1.26–5.75	0.01	3.17	1.29–7.81	0.01
Paracetamol etiology	2.17	1.01–4.68	0.05	2.50	1.00–6.28	0.05
Age (year)	0.99	0.96–1.01	0.29			

Among listed patients, 1‐year and 5‐year survival rates were both 74% (64–87%). Among those not listed, survival rates were 33% (22–48%) and 28% (17–46%), respectively.

#### *Other diagnostic categories*

For biliary cirrhosis/sclerosing cholangitis, 1‐year and long‐term survival rates were identical. Survival rates were 90% (73–100%) among patients who were listed and 67% (38–100%) among those not listed.

Among transplant patients, when listed for retransplant, the survival rates at both hospital discharge and long‐term follow‐up were 80% in case of nonfunction after LTx, and hospital, 1‐year, and 5‐year survival rates were 67% (38–88%), 72% (54–96%), and 60% (0.40–0.88), respectively, in case of late graft dysfunction. In contrast, 1‐year survival was 0% in both categories when patients were not listed (all *P* < 0.001). None of the patients with liver failure after nontransplant liver surgery were listed, and none were alive at 1‐year follow‐up. Patients with other liver diseases had identical 1‐year and long‐term survival rates: 75% (43–100%) when listed and 57% (35–94%) when not listed (*P* = 0.51). Regarding other diagnoses, only one patient was listed (alive at the 3‐week follow‐up), and the other 15 had identical 1‐year and 4‐year survival rates of 38% (20–77%).

#### *Comparison between pre‐MELD and post‐MELD periods*

We compared long‐term survival between the first study period (pre‐MELD, 2004–March 2007, when the graft allocation system was not based on the MELD score, resulting in a prolonged waiting time on the transplant list) and the second period (MELD‐based, March 2007–2009). Diagnoses (*P* = 0.47) and percentages on the waiting list (31.8% and 38.1%, *P* = 0.19) did not differ significantly between periods. The relative risk of death did not change significantly between the two periods (*P* > 0.30 for each diagnostic category).

## Discussion

In this large cohort of patients, the indications for MARS therapy were not restricted, and only a minority of patients was included in a clinical trial. Major hyperbilirubinemia (total bilirubin >340 μmol/L), HE of grade 2 or more, and kidney dysfunction were three major indications among patients with cirrhosis or other pre‐existing chronic liver diseases. High survival rates were shown mainly for the patients listed for transplant. Two randomized controlled trials published in 2013 did not report any significant improvement in transplant‐free survival.^9^, ^10^ This suggests that the use of MARS in these indications should be limited to patients with a transplant project.

MARS for HE has been used a rescue therapy for patients refractory to medical treatment. The benefit on the clinical HE grade and a favorable effect on the amino acid profile have been reported in several randomized trials.^5^, ^6^, ^11^ MARS has been commonly used to treat AKI, although its direct effect on HRS is controversial. Because the MARS monitor is combined with a hemodialysis or hemodiafiltration generator, repeated MARS sessions are able to provide an adequate dialysis dose.^12^, ^13^

MARS therapy for cholestasis refractory pruritus, a less frequent but well‐defined indication, was associated with fairly good hospital survival. Most patients have an immediate complete or partial response.^14^

ALF emerged as a well‐defined indication for MARS therapy in one third of cases. MARS treatment has been proposed to sustain the patient until a graft becomes available or the native liver regenerates.^15^ More rarely, MARS has been used to treat early allograft dysfunction after LTx^16^ or liver failure after hepatectomy.^17^ These two indications each accounted for 9.9% of treatments in our study.

MARS albumin dialysis is able to remove a number of albumin‐bound drugs. However, the indication for drug overdose was rare in our study.

Of patients, 66% were not on the transplant list, suggesting that MARS was mostly used as a bridge to improvement for patients with chronic advanced liver disease (nonbiliary cirrhosis: 74%; late graft dysfunction: 62%) or as a bridge to recovery in ALF (57%). Indeed, the criteria for listing in emergency ALF patients for transplantation with the “High‐emergency priority rule” did no not change in the last two decades in France.

Among patients with chronic liver disease, the proportion of patients on the list in our study was higher than usually reported. In comparison, in the CANONIC study, the proportion of listed patients was only 13.5%, and 4.1% were transplanted by day 28.^18^ Among ALF patients, the proportion of patients listed was similar to that (38%) reported in the U.S. cohort 1998–2013.^19^

In nonbiliary cirrhosis, we found that hyperbilirubinemia was associated with a high risk of hospital death. The increase in bilirubin increases the MELD score, which predicts 90‐day mortality among patients waiting for LTx,^20^ and could contribute to renal toxicity.^21^ HE was also identified as a predictor of hospital death in our study, although MARS therapy can improve HE. This finding is in accordance with other reports.^9^, ^22^ Among patients who have not been listed, the 1‐year survival rate was particularly low for nonbiliary cirrhosis (15%), suggesting the futility of MARS therapy in this setting. In contrast, listing, followed by early transplantation, among severely ill cirrhotic patients with multiple organ dysfunctions has been recently associated with good outcomes.^23^

Regarding the patients with either nonfunction after LTx or chronic graft dysfunction, the 0% survival rate among those not listed for retransplantation does not support the use of MARS as a salvage therapy.

In ALF, a sizeable proportion (45%) of the patients who were not listed were alive at hospital discharge. Analysis of ALF patients' outcome mostly focused on hospital survival because the patients who recovered during their hospital stay and survived without LTx did not require long‐term follow‐up. Paracetamol etiology and number of sessions ≥3 were associated with better transplant‐free survival, in accordance with the results of the FULMAR study.^10^ Whether MARS could improve survival^24^ or reduce the need for LTx^25^, ^26^ remains debated, and no definite recommendation can be made.

This study has some limitations related to the retrospective design and data collection and the use of MARS among patients with end‐stage liver disease and multiple organ failure.

In conclusion, this is the largest multicenter study collecting data on the use of MARS in real life and following patients in the long term. Survival was correlated with being listed for transplant in all etiologies and with the intensity of treatment in ALF. The study has established the main determinants of poor prognosis in patients with decompensated nonbiliary cirrhosis and has highlighted the need to use MARS mainly as a bridge to LTx for all patients.

## Supporting information


**Table S1**. Transplantation during hospital stay for patients on the waiting list.
**Table S2**. Reported indications for MARS treatments.
**Table S3**. Follow‐up after hospital discharge according to the main diagnosis.
**Table S4**. Variables associated with hospital spontaneous survival in patients with acute liver failure and a hospital length of stay ≥4 days.
