# Supplementary material for: Outcome of patients treated with molecular adsorbent recirculating system albumin dialysis: A national multicenter study
Source: JGH Open. 2020 May 17;4(4):757–63. doi: 10.1002/jgh3.12359 (PMC7411551; doi:10.1002/jgh3.12359)
Supplement: Supplementary file 1 — Table S1. Transplantation during hospital stay for patients on the waiting list. Table S2. Reported indications for MARS treatments. Table S3. Follow‐up after hospital discharge according to the main diagnosis. Table S4. Variables associated with hospital spontaneous survival in patients with acute liver failure and a hospital length of stay ≥4 days. [file JGH3-4-757-s001.docx]

Supporting information.

Table 1. Transplantation during the hospital stay in patients on the waiting list.

|  | No. transplanted/no. listed (%) |
| --- | --- |
| Preexisting liver disease / liver transplantation  Non-biliary cirrhosis | 48/78 (64.9)  19/36 (52.8) |
| Biliary cirrhosis / sclerosing cholangitis | 7/8 (87.5) |
| Other chronic liver disease | 2/5 (40.0) |
| Post-transplant surgery  Non-function after liver transplantation  Late graft dysfunction | 20/25 (80.0)  8/10 (80.0)  12/15 (80.0) |
| Non-transplant liver surgery |  |
| Acute liver failure | 38/56 (67.9) |
| Other  Total | 0/1  86/131 (65.6) |

Table2. Reported indications for MARS treatments.

|  | Total | Non-biliary cirrhosis | | Biliary cirrhosis/sclerosing cholangitis | | Other chronic liver disease | | Non-function after LTx | | Late graft dysfunction | | Liver failure after non-LTx liver surgery | | Acute liver failure | | Other | |
| --- | --- | --- | --- | --- | --- | --- | --- | --- | --- | --- | --- | --- | --- | --- | --- | --- | --- |
|  | N=393 | Not listed  N=101 | Listed  N=37 | Not listed  N=8 | Listed  N=8 | Not listed  N=16 | Listed  N=5 | Not listed  N=9 | Listed  N=11 | Not listed  N=24 | Listed  N=17 | Not listed  N=12 | Listed  N=0 | Not listed  N=73 | Listed  N=56 | Not listed  N=15 | Listed  N=1 |
| Indication  No. (percent [95% confidence interval]) |  |  |  |  |  |  |  |  |  |  |  |  |  |  |  |  |  |
| Major hyperbilirubinemia | 146 | 65 (64.4 [54.2-73.6]) | 20 (54.1 [36.9-70.5]) | 2 (25.0 [3.2-65.1]) | 8 (100 [65.1-100]) | 2 (12.5 [1.6-38.6]) | 3 (60.0 [14.7-94.7]) | 1 (11.1 [0.3-48.3]) | 1 (9.1 [0.2-41.3]) | 18 (75.0 [54.3-90.4]) | 11 (64.7 [38.3-85.8]) | 6 (50.0 [21.1-78.9]) |  | 4 (5.5 [1.5-13.4]) | 2 (3.6 [0.4-12.3]) | 2 (13.3 [1.7-40.5]) | 1 (100 [3.0-100]) |
| Hepatic encephalopathy | 93 | 45 (44.6 [34.7-54.8]) | 15 (40.5 [24.8-57.9] ) | 1 (12.5 [0.3-52.7]) | 5 (62.5 (24.5-91.5]) | 2 (12.5 [1.6-38.6]) | 1 (20.0 [0.5-71.6]) | 1 (11.1 [0.3-48.3]) |  | 10 (41.7 [22.1-63.4]) | 6 (35.3 [14.2-61.7]) | 3 (25.0 [5.5-57.2]) |  | 2 (2.7 [0.3-9.6]) | 2 (3.6 [0.4-12.3]) |  |  |
| Hepatorenal syndrome | 60 | 35 (34.7 [25.5-44.8]) | 10 (27.0 [13.8-44.1]) | 1 (12.5 [0.3-52.7]) | 1 (12.5 [0.3-52.7]) | 2 (12.5 [1.6-38.6]) |  |  | 1 (9.1 [0.2-41.3]) | 4 (16.7 [4.8-37.4]) | 3 (17.6 [3.8-43.4]) | 2 (16.7 [2.1-48.4]) |  |  | 1 (1.8 [0.1-9.6]) |  |  |
| Other kidney dysfunction | 30 | 8 (7.9 [3.5-15.0]) | 4 (10.8 [3.0-25.4]) |  | 1 (12.5 [0.3-52.7]) | 1 (6.3 [0.2-30.2]) |  | 1 (11.1 [0.3-48.3]) |  | 4 (16.7 [4.8-37.4]) | 4 (23.5 [6.8-49.9]) |  |  | 4 (5.5 [1.5-13.4]) | 2 (3.6 [0.4-12.3]) | 1 (6.7 [0.2-32.0]) |  |
| Cholestasis refractory pruritus | 39 | 2 (2.0 [0.2-7.0]) | 1 (2.7 [0.1-14.2]) | 7 (87.5) | 1 (12.5 [0.3-52.7]) | 8 (50.0 [24.7-75.4]) | 4 (80.0 [7.3-52.4]) |  |  | 9 (37.5 [18.8-59.4]) | 5 (29.4 [10.3-56.0]) |  |  |  |  | 1 (6.7 [0.2-32.0]) | 1 (100 [3.0-100]) |
| Acute alcoholic hepatitis | 49 | 39 (38.6 [29.1-48.8]) | 10 (27.0 [13.8-44.1]) |  |  |  |  |  |  |  |  |  |  |  |  |  |  |
| ALF† | 128 |  | 1 (2.7 [0.1-14.2]) |  |  |  |  |  |  |  |  |  |  | 72 (98.6 [92.6-100.0]) | 55 (98.2 [90.5-100.0]) |  |  |
| ALF after LTx | 24 |  |  |  |  |  |  | 9 (100 [66.4-100]) | 11 (100 [71.5-100]) | 3 (12.5 [2.7-32.4]) | 1 (5.9 [0.2-28.7]) |  |  |  |  |  |  |
| ALF after liver resection | 15 |  |  |  |  | 2 (12.5 [1.6-38.6]) |  |  |  |  |  | 11 (91.7 [61.5-99.8]) |  |  | 1 (1.8 [0.1-9.6]) | 1 (6.7 [0.2-32.0]) |  |
| Drug intoxication | 6 |  |  |  |  |  |  |  |  |  |  |  |  |  |  | 6 (40.0 [16.3-67.7]) |  |
| Other | 20 | 8 (7.9 [3.5-15.0]) | 1 (2.7 [0.1-14.2]) |  | 1 (12.5 [0.3-52.7]) | 4 (25.0 [7.3-52.4]) |  | 1 (11.1 [0.3-48.3]) |  |  |  |  |  |  |  | 5 (33.3 [11.8-61.6]) |  |
| ≥2 indications | 146 | 59 (58.4 [48.2-68.1]) | 18 (48.6 [31.9-65.6]) | 2 (25.0 [3.2-65.1]) | 8 (100 [65.1-100]) | 3 (18.8 [4.1-45.7]) | 3 (60.0 [14.7-94.7]) | 3 (33.3 [7.5-70.1]) | 1 (9.1 [0.2-41.3]) | 20 (83.3 [62.6-95.3]) | 12 (70.6 [44.0-89.7]) | 6 (50.0 [21.1-78.9]) |  | 6 (8.2 [3.1-17.0]) | 3 (5.4 [1.1-14.9]) | 1 (6.7 [0.2-32.0]) | 1 (100 [3.0-100]) |

LTx, liver transplantation ; †ALF, acute liver failure; indication”ALF “at the time of MARS treatment could be different from the final diagnosis of ALF.

Table 3. Follow-up after hospital discharge according to the main diagnosis.

|  | Hospital death | No follow-up | Follow-up | |
| --- | --- | --- | --- | --- |
|  | No. of patients | No. of patients | No. of patients | Duration, days |
| Diagnosis  Non-biliary cirrhosis | 85 | 10 | 42 | 714 [248‒1202] |
| Biliary cirrhosis/sclerosing  cholangitis | 3 | 1 | 12 | 1112 [813‒1308] |
| Other chronic liver disease | 6 | 2 | 8 | 423 [211‒1159] |
| Nonfunction after LTx | 11 | 0 | 8 | 837 [647‒1957] |
| Late graft dysfunction | 22 | 1 | 13 | 766 [610‒1003] |
| Liver failure after non-transplant liver surgery | 9 | 3 | 0 | ‒ |
| Acute liver failure | 54 | 14 | 61 | 931 [351‒1569] |
| Other | 8 | 1 | 7 | 293 [52‒808] |
| Total patients | 198 | 32 | 153 | 812 [321‒1373] |

Note. Durations are medians (25^th^ percentile –75^th^ percentile). Among the 32 patients who were lost to follow-up, 14 were not candidates for transplantation due to the absence of alcohol abstinence (7), the existence of liver cancer beyond transplant criteria (4) or other reasons (3). Fourteen patients recovered from ALF during their hospital stay. Three other patients, who were treated for refractory pruritus, were not considered to have severe liver disease. The last patient was transplanted and lost to follow-up after hospital discharge.

Table 4. Variables associated with hospital spontaneous survival in patients with acute liver failure and a hospital length of stay ≥4 days.

| Variable | Univariate | | | Multivariate | | |
| --- | --- | --- | --- | --- | --- | --- |
|  | Odds ratio | 95%CI | P-value | Odds ratio | 95%CI | P-value |
| Listed with the “High emergency” priority | 0.22 | 0.09-0.53 | <0.001 | 0.23 | 0.09-0.59 | 0.002 |
| ≥3 MARS sessions | 2.42 | 1.12-5.34 | 0.02 | 2.71 | 1.08-6.80 | 0.03 |
| Paracetamol aetiology | 2.96 | 1.32-6.67 | 0.009 | 3.47 | 1.33-9.07 | 0.01 |
| Age (per increasing year) | 0.99 | 0.96-1.01 | 0.20 |  |  |  |

NOTE. MARS: molecular adsorbent recirculating system.
